# Supplementary material for: The role of nucleobase interactions in RNA structure and dynamics
Source: Nucleic Acids Res. 2014 Oct 29;42(21):13306–14. doi: 10.1093/nar/gku972 (PMC4245972; doi:10.1093/nar/gku972)
Supplement: SUPPLEMENTARY DATA [file supp_42_21_13306__index.html]

The role of nucleobase interactions in RNA structure and dynamics — SUPPLEMENTARY DATA 

# The role of nucleobase interactions in RNA structure and dynamics

## SUPPLEMENTARY DATA

**Files in this Data Supplement:**

- SUPPLEMENTARY DATA
