# Supplementary material for: The role of nucleobase interactions in RNA structure and dynamics
Source: Nucleic Acids Res. 2014 Oct 29;42(21):13306–14. doi: 10.1093/nar/gku972 (PMC4245972; doi:10.1093/nar/gku972)
Supplement: SUPPLEMENTARY DATA [file supp_gku972_nar-02117-r-2014-File002.zip › supporting/supporting.pdf]

The Role of Nucleobase Interactions in RNA Structure and Dynamics

Sandro Bottaro, Francesco di Palma and Giovanni Bussi

Supplementary Data

- **Figure SD1** θ/ρ probability density distributions in the pairing and stacking zone obtained from a non-redundant PDB dataset.
- **Figure SD2** θ/ρ probability density distributions in the paring zone for the 16 different nucleotide combinations.
- **Figure SD3** θ/ρ probability density distributions in the stacking zone for the 16 different nucleotide combinations.
- **Text SD4** Description of the non-linear mapping **G** for $\mathcal{E}$RMSD calculation.
- **Figure SD5** Comparison between $\mathcal{E}$RMSD and the scalar version of $\mathcal{E}$RMSD.
- **Table SD6** Summary of the decoy screening capabilities of different scoring functions.
- **Figure SD7** $\mathcal{E}$SCORE versus RMSD plots of the FARNA decoy sets [1].
- **Figure SD8** $\mathcal{E}$SCORE versus RMSD plots of the NM and MD decoy sets [2].
- **Text SD9** Molecular dynamics simulation methods.
- **Text SD10** Description of the motif search strategy.

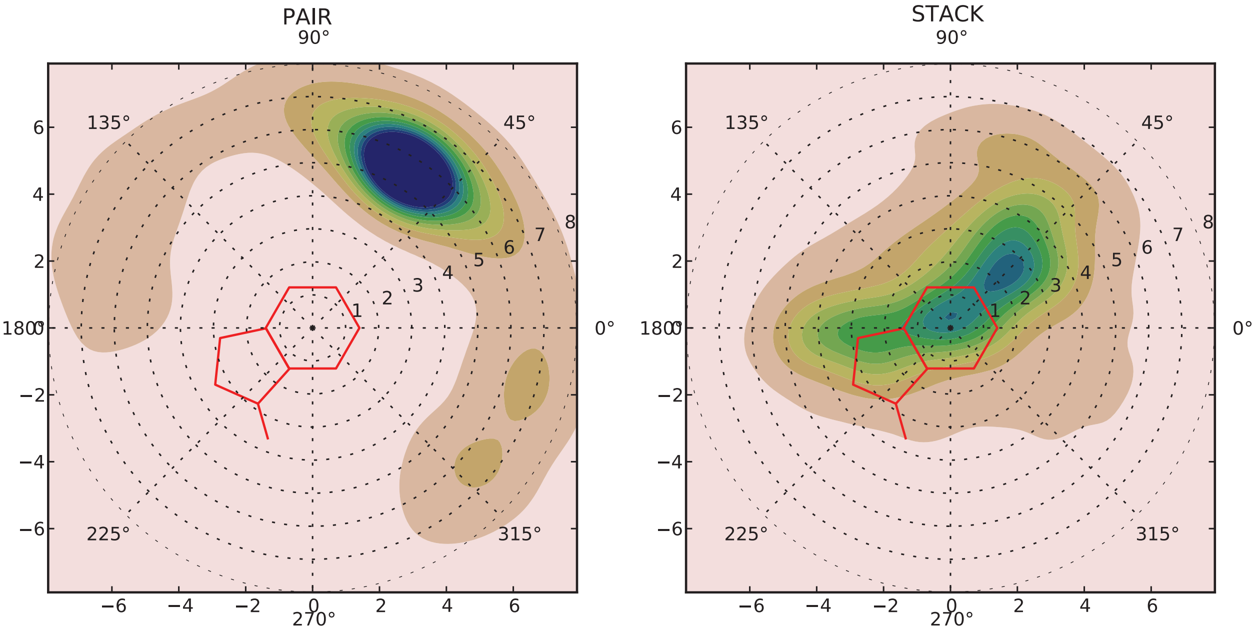

Figure SD1. Empirical density distributions of neighboring nucleobases obtained by projecting points belonging to the pairing and stacking zone on the $\theta - \rho$ plane. At variance with Figure 1 in main text, the plots are obtained using a high-resolution, non-redundant RNA dataset[2]. As a reference, the 6-membered and 5-membered (for purines only) rings are sketched in red. Plots were obtained using a Gaussian kernel density estimation with bandwidth=0.25Å.

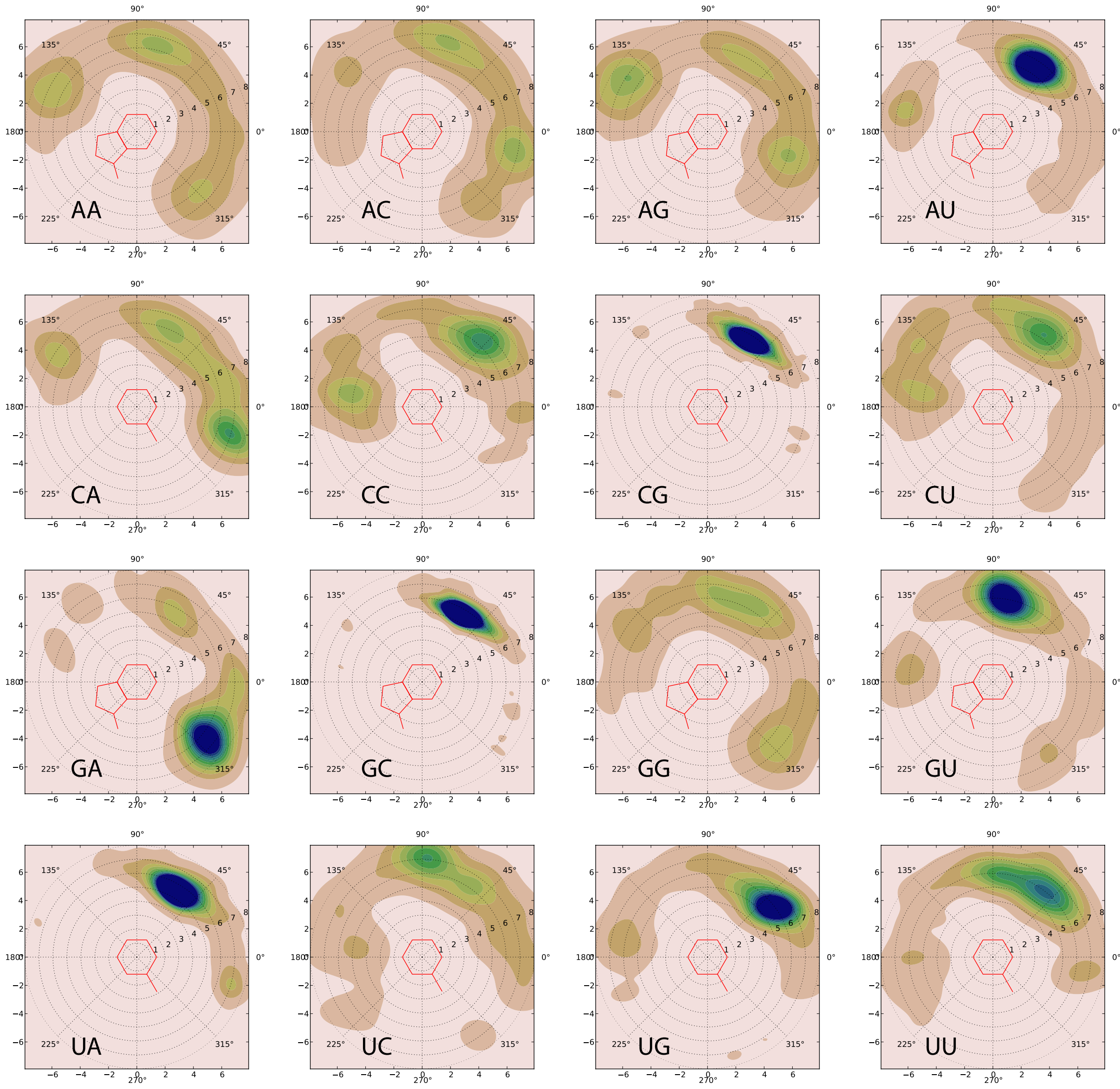

Figure SD2. Empirical density distributions of neighboring nucleobases obtained by projecting points belonging to the pairing zone on the $\theta - \rho$ plane. All the 16 possible combinations between nucleotides are reported, as labeled. As a reference, the 6-membered and 5-membered (for purines only) rings are sketched in red. Plots were obtained using a Gaussian kernel density estimation with bandwidth=0.25Å.

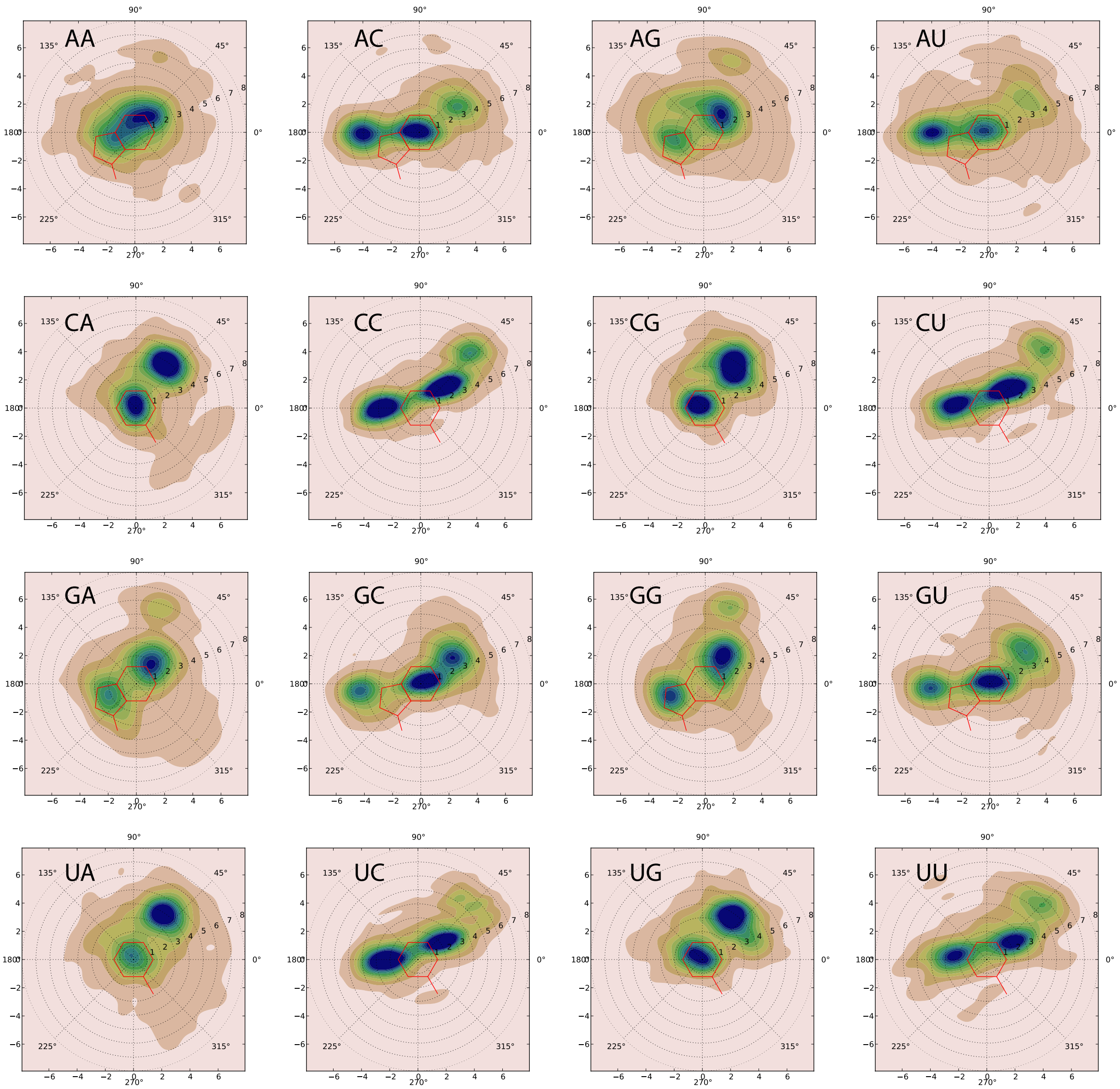

Figure SD3. Empirical density distributions of neighboring nucleobases, obtained by projecting points belonging to the stacking zone on the $\theta - \rho$ plane. All the 16 possible combinations between nucleotides are reported, as labeled. As a reference, the 6-membered and 5-membered (for purines only) rings are sketched in red.

Text SD4.

The relationship between distances in $\mathbf{G}$ space and distances in $\tilde{\mathbf{r}}$ space is important to understand how $\mathcal{E}$RMSD discriminates between two structures α and β depending on the similarity of the contacts formed in the two structures. Here, we discuss three possible cases:

- $\tilde{r}^\alpha \geq \tilde{r}_{\text{cutoff}}, \tilde{r}^\beta \geq \tilde{r}_{\text{cutoff}}$
  In the case of a vector $\tilde{\mathbf{r}}$ with modulus $\tilde{r} \geq \tilde{r}_{\text{cutoff}}$ the corresponding $\mathbf{G}(\tilde{\mathbf{r}})$ vector is the null vector. Thus, $\tilde{\mathbf{r}}$ vectors larger than the cutoff distance are considered as equivalent. This implies that if a contact between two bases is not formed in structures α and β, this contact does not contribute to the $\mathcal{E}$RMSD between the two structures.
- $\tilde{r}^\alpha \geq \tilde{r}_{\text{cutoff}}, \tilde{r}^\beta < \tilde{r}_{\text{cutoff}}$
  In this case the modulus of the distance in $\mathbf{G}$ space reduces to $|\mathbf{G}(\tilde{\mathbf{r}}^\beta) - \mathbf{G}(\tilde{\mathbf{r}}^\alpha)| = |\mathbf{G}(\tilde{\mathbf{r}}^\beta)| = \frac{1}{\gamma}\sqrt{2 + 2\cos\gamma\tilde{r}^\beta} = \frac{2}{\gamma}\cos\frac{\gamma}{2}\tilde{r}^\beta$. As a consequence, the contribution to the $\mathcal{E}$RMSD of a contact that is formed in one of the two structures and not in the other goes smoothly to zero as $\tilde{r}^\beta$ approaches $\tilde{r}_{\text{cutoff}}$. This is the desired behavior of a smooth contact map.
- $\tilde{r}^\alpha < \tilde{r}_{\text{cutoff}}, \tilde{r}^\beta < \tilde{r}_{\text{cutoff}}$
  The case where a contact is formed in both structures is more complicated. This contact will contribute to the $\mathcal{E}$RMSD depending on how different are the distances and the approach angles. The angular dependence vanishes when the contact distance approaches the cutoff value, as discussed below.

We first consider the case of two vectors $\tilde{\mathbf{r}}^\alpha$ and $\tilde{\mathbf{r}}^\beta$ with *different modulus and identical direction* ($\frac{\tilde{\mathbf{r}}^\alpha}{\tilde{r}^\alpha} = \frac{\tilde{\mathbf{r}}^\beta}{\tilde{r}^\beta} \equiv \frac{\tilde{\mathbf{r}}}{\tilde{r}}$). Their difference is

$$\Delta\mathbf{G} = \frac{1}{\gamma}\begin{pmatrix} (\sin\gamma\tilde{r}^\beta - \sin\gamma\tilde{r}^\alpha)\frac{\tilde{r}_x}{\tilde{r}} \\ (\sin\gamma\tilde{r}^\beta - \sin\gamma\tilde{r}^\alpha)\frac{\tilde{r}_y}{\tilde{r}} \\ (\sin\gamma\tilde{r}^\beta - \sin\gamma\tilde{r}^\alpha)\frac{\tilde{r}_z}{\tilde{r}} \\ \cos\gamma\tilde{r}^\beta - \cos\gamma\tilde{r}^\alpha \end{pmatrix}$$

After proper algebra it can be shown that the modulus of this difference is $|\Delta\mathbf{G}| = \frac{1}{\gamma}\sqrt{2 - 2\cos\gamma(\tilde{r}^\beta - \tilde{r}^\alpha)} = \frac{2}{\gamma}\sin\frac{\gamma}{2}|\tilde{r}^\beta - \tilde{r}^\alpha|$. If the two moduli $\tilde{r}^\alpha$ and $\tilde{r}^\beta$ are close to each other then the expression above can be approximated to first order as $|\Delta\mathbf{G}| \approx \left|\tilde{r}^\beta - \tilde{r}^\alpha\right|$. As a consequence, if two vectors $\tilde{\mathbf{r}}$ are close to each other and pointing in the same direction, their distance in $\mathbf{G}$ space is equal to their distance in $\tilde{\mathbf{r}}$ space. This property also holds for the simplified scalar distance based on Eq. 6 in main text.

We then consider the case of two vectors $\tilde{\mathbf{r}}^\alpha$ and $\tilde{\mathbf{r}}^\beta$ with *identical modulus and different direction* ($\tilde{r}^\alpha = \tilde{r}^\beta \equiv \tilde{r}$). Their difference is

$$\Delta\mathbf{G} = \frac{\sin\gamma\tilde{r}}{\gamma\tilde{r}}\begin{pmatrix} \tilde{r}_x^\beta - \tilde{r}_x^\alpha \\ \tilde{r}_x^\beta - \tilde{r}_y^\alpha \\ \tilde{r}_x^\beta - \tilde{r}_z^\alpha \\ 0 \end{pmatrix}$$

This expression implies $|\Delta\mathbf{G}| = \frac{\sin\gamma\tilde{r}}{\gamma\tilde{r}}\left|\tilde{\mathbf{r}}^\beta - \tilde{\mathbf{r}}^\alpha\right|$. As a consequence, if two vectors $\tilde{\mathbf{r}}$ have the same modulus, their distance in $\mathbf{G}$ space is scaled by a modulus-dependent factor when compared with the distance in $\tilde{\mathbf{r}}$ space. The function $\frac{\sin\gamma\tilde{r}}{\gamma\tilde{r}}$ interpolates between 1 (for $\tilde{r} \to 0$) and 0 (for $\tilde{r} = \tilde{r}_{\text{cutoff}}$) so that the weight of the angular distance decreases as the modulus approaches $\tilde{r}_{\text{cutoff}}$. This is necessary to have a continuous function, since the distance of two vectors $\tilde{\mathbf{r}}$ for which $\tilde{r} = \tilde{r}_{\text{cutoff}}$ should be zero. This angular contribution is ignored in the simplified scalar distance based on Eq. 6 in main text.

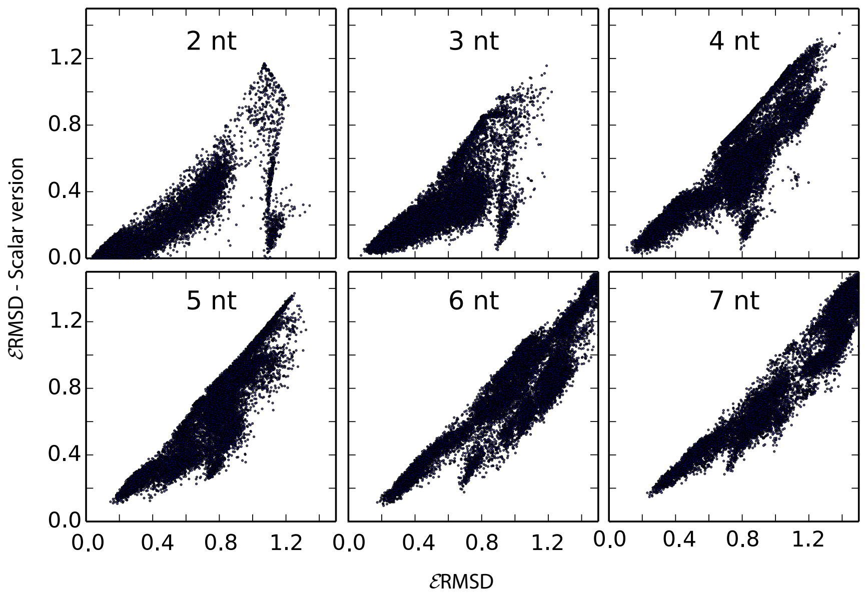

Figure SD5. Comparison between $\mathcal{E}$RMSD (Eq. 4, main text) and the scalar version (Eq. 6). When considering distances between structures composed by two, three and 4 nucleotides, the two quantities are poorly correlated. Structures were obtained from a steered molecular dynamics simulation of a short hairpin loop (See Fig. 4 in main text and Text SD9).

SET	PDB	Ranking			RMSD (Å)			INF			$\mathcal{E}RMSD$		
		$\mathcal{E}$SCORE	FARFAR	RASP	$\mathcal{E}$SCORE	FARFAR	RASP	$\mathcal{E}$SCORE	FARFAR	RASP	$\mathcal{E}$SCORE	FARFAR	RASP
NM	1ykq	0.22	0.00	0.00	1.45	3.46	1.74	0.79	0.77	0.79	0.66	0.82	0.64
NM	1zih	0.00	0.04	0.05	1.42	1.81	1.28	0.84	0.76	0.78	0.50	0.73	0.50
NM	28sp	0.00	1.00	0.00	1.77	4.80	1.42	0.76	0.67	0.71	0.65	1.14	0.66
NM	2f88	0.00	0.00	0.00	1.85	2.34	1.85	0.79	0.78	0.79	0.50	0.53	0.50
NM	434d	0.00	0.00	0.00	1.35	3.10	1.38	0.90	0.55	0.90	0.29	1.08	0.36
NM	1duq	0.00	0.00	0.00	1.24	2.42	1.19	0.93	0.77	0.91	0.38	0.79	0.36
NM	1esy	0.00	0.28	0.02	1.39	2.06	1.39	0.80	0.73	0.80	0.56	0.65	0.56
NM	1f27	0.00	0.00	0.00	1.34	4.53	1.33	0.89	0.72	0.89	0.54	1.20	0.53
NM	1i9v	0.00	0.00	0.00	2.37	3.93	2.41	0.82	0.68	0.80	0.58	1.08	0.59
NM	1kka	0.16	0.30	0.16	1.16	1.30	1.69	0.70	0.66	0.83	0.66	0.91	0.69
NM	1msy	0.00	0.00	0.00	2.18	4.59	1.50	0.72	0.71	0.71	0.67	1.09	0.57
NM	1nuj	0.00	0.00	0.00	1.75	4.63	1.56	0.89	0.77	0.86	0.62	1.13	0.67
NM	1qwa	0.00	0.01	0.03	1.55	1.09	1.94	0.49	0.59	0.67	0.75	0.68	0.79
NM	1xjr	0.00	0.00	0.00	1.28	4.32	1.15	0.84	0.65	0.81	0.45	1.03	0.43
MD	1duq	0.00	0.00	0.00	0.32	0.42	0.32	0.93	0.94	0.94	0.19	0.25	0.23
MD	1f27	0.00	0.00	0.00	1.28	0.40	1.24	0.91	0.92	0.93	0.44	0.22	0.34
MD	1msy	0.00	0.00	0.01	0.38	2.23	0.43	0.92	0.80	0.87	0.19	0.57	0.25
MD	1nuj	0.00	0.00	0.01	0.73	0.73	2.88	0.92	0.93	0.66	0.49	0.49	0.97
MD	434d	0.00	0.00	0.00	0.28	0.30	0.29	0.90	0.92	0.95	0.19	0.19	0.23
FARNA	157d	0.00	0.00	0.00	3.54	3.03	2.14	0.80	0.62	0.72	0.65	1.09	0.93
FARNA	1a4d	0.00	0.00	0.01	23.57	23.87	6.40	0.41	0.38	0.56	2.23	2.19	1.14
FARNA	1csl	0.00	0.00	0.00	3.96	3.35	4.42	0.63	0.75	0.71	1.29	1.16	1.33
FARNA	1dqf	0.00	0.00	0.00	3.04	3.11	3.21	0.90	0.78	0.90	0.81	0.89	0.89
FARNA	1esy	0.00	0.14	0.56	3.70	3.99	3.16	0.51	0.54	0.67	1.28	1.38	1.22
FARNA	1i9x	0.00	0.00	0.00	4.79	5.18	4.79	0.82	0.72	0.82	1.11	1.17	1.11
FARNA	1j6s	0.00	0.00	0.03	10.66	12.94	12.81	0.51	0.35	0.37	2.29	2.29	2.37
FARNA	1kd5	0.00	0.00	0.01	4.22	4.81	4.08	0.70	0.56	0.65	1.30	1.40	1.30
FARNA	1kka	0.30	0.28	1.00	4.15	5.65	4.98	0.70	0.65	0.73	1.04	1.11	1.07
FARNA	1l2x	0.00	0.00	0.37	13.62	8.23	15.76	0.44	0.38	0.40	2.24	1.92	2.32
FARNA	1mhk	0.00	0.00	0.00	9.03	9.37	8.89	0.71	0.70	0.65	1.42	1.26	1.42
FARNA	1q9a	0.00	0.00	0.91	4.74	5.61	5.56	0.53	0.61	0.61	1.28	1.27	1.24
FARNA	1qwa	0.00	0.24	1.00	4.24	4.51	4.93	0.48	0.53	0.38	1.38	1.20	1.50
FARNA	1xjr	0.00	0.00	0.03	8.81	9.11	9.66	0.15	0.13	0.12	1.70	1.61	1.63
FARNA	1zih	0.00	0.10	0.35	1.84	1.93	1.99	0.90	0.69	0.90	0.58	0.68	0.58
FARNA	255d	0.00	0.00	0.11	1.90	1.99	2.93	0.79	0.78	0.80	0.55	0.54	0.63
FARNA	283d	0.00	0.00	0.03	3.12	3.67	2.77	0.78	0.66	0.77	0.92	0.96	0.88
FARNA	28sp	0.00	0.96	0.51	3.73	3.18	3.64	0.69	0.68	0.65	1.19	1.20	1.40
FARNA	2a43	0.00	0.00	0.19	4.78	5.06	5.34	0.52	0.39	0.48	1.81	1.87	1.86
FARNA	2f88	0.00	0.00	0.23	3.87	6.54	4.36	0.69	0.56	0.63	0.96	1.36	1.33

Table SD6. Performance of $\mathcal{E}$SCORE compared to the all-atom scoring functions FARFAR and RASP. For each decoy set, we report the normalized rank and the deviation of the best scoring decoy from the native structure. Structural deviation is calculated with the standard RMSD measure, as well as using the interaction fidelity network (INF) and the $\mathcal{E}$RMSD. The best-performing scoring function(s) are highlighted in green for each decoy set and for each measure. Note that a good normalized rank does not necessarily imply a good performance in the decoy screening test.

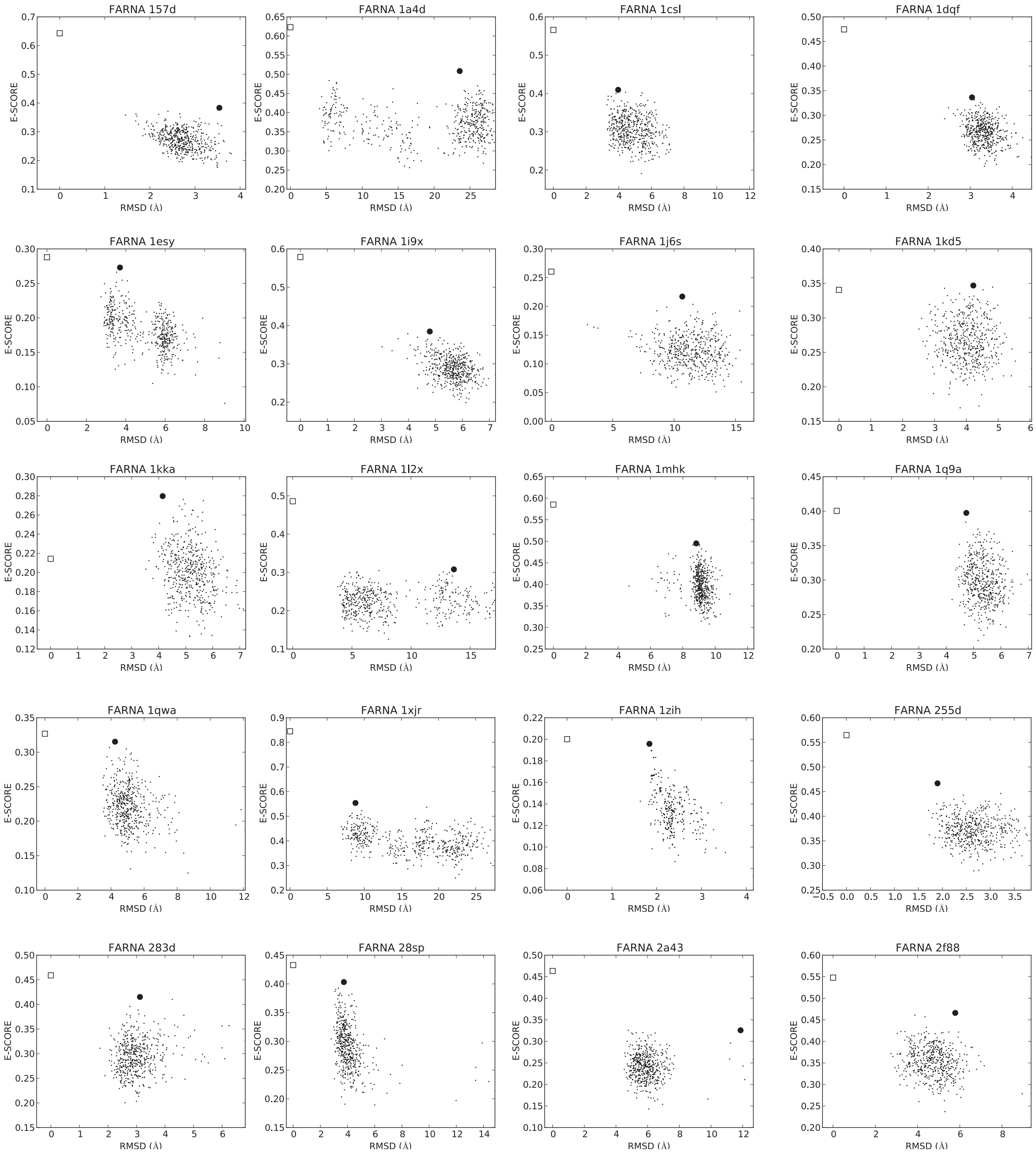

Figure SD7. RMSD versus $\mathcal{E}$SCORE for the 20 decoy sets generated using the FARNA algorithm (`http://daslab.stanford.edu/das_resources.html`) [1]. The white square indicates the score of the native structure, while the best scoring decoy is shown as a black circle. Notice that for most of the decoy sets there is a poor correlation between the RMSD from native and the $\mathcal{E}$SCORE. Notable examples include the decoy sets 1A4D, 1L2X and 2A43, for which the $\mathcal{E}$SCORE is able to discriminate the native structure (normalized rank=0), but fails to identify the best scoring structure within the decoy set.

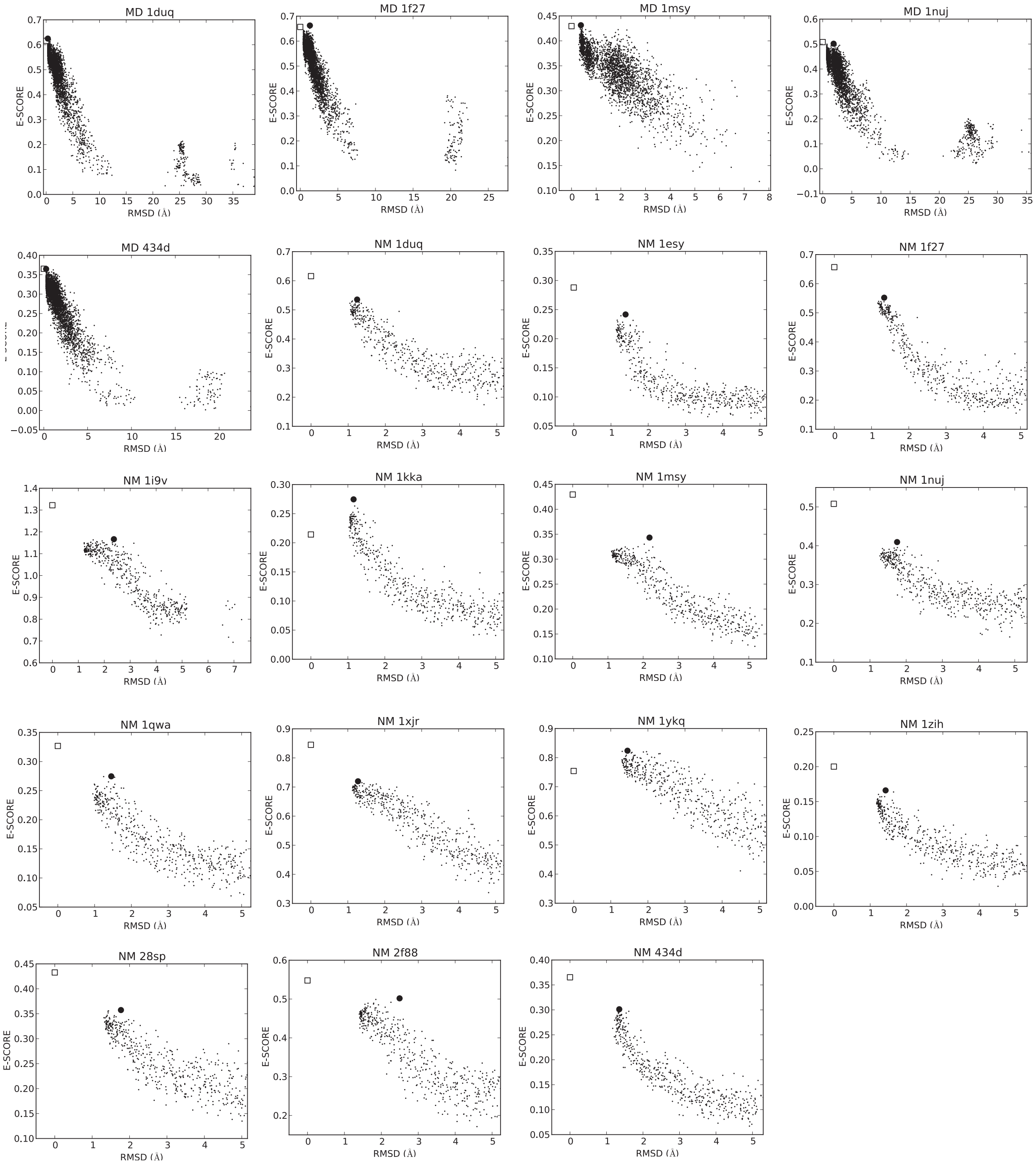

Figure SD8. RMSD versus $\mathcal{E}$SCORE for 19 decoy sets obtained from the work of Bernauer et al. [2] (`http://csb.stanford.edu/rna/download.html`). The white square indicates the score of the native structure, while the best scoring decoy is shown as a black circle. The decoy set NM 1X9K was discarded as the decoy sequence differs from the native one. Notice that there is a clear correlation between the RMSD from native and the $\mathcal{E}$SCORE.

Text SD9. All simulations were performed using GROMACS 4.5 [3] and PLUMED 2.0 [4] with the AMBER99sb force field [5] and parambsc0 [6] + chiOL [7] corrections. All simulations were performed in NPT ensemble (T=300K, P=1 atm) with stochastic velocity rescaling [8] and Berendsen barostat [9]. Long range electrostatics were treated using Particle-Mesh Ewald summation [10]. The equations of motion were integrated with a 2 fs time step. All bond lengths were constrained using the LINCS algorithm [11].

Steered molecular dynamics. A gccUUCGggc stem-loop (residues 6-15 from PDB code 1F7Y [12]) was solvated with 2627 TIP3P [13] water molecules and NaCl at 0.1 M in a rhombic dodecahedral box. A 85ns steered molecular dynamics simulation was performed using as a collective variable the RMSD from the native structure. Starting from the native structure, the unfolding/folding was simulated by repeatedly applying an harmonic restraint from 0 to 1 nm (and vice versa) with speed $0.1nm/ns$ and force constant $k = 3000kJmol^{-1}nm^{-2}$. INF measure was calculated as described in Ref.[14], considering base-pairing as well as stacking, base-phosphate and base-sugar interactions.

Free molecular dynamics of *add* Riboswitch The kinetic analysis presented in Fig.5 was performed on a 100ns simulation of the *add* riboswitch [15]. The crystal structure was solvated with 11190 water molecules and NaCl at 0.1 M in a rhombic dodecahedral box, and ligand was removed. RMSD and dRMSD were calculated using the GROMACS software package.

Text SD10. The internal loop motif search was performed as follows:

- Obtain the query motif by extracting from the PDB file the atomic coordinates of the nucleotides composing the motif. A double stranded motif of length n is composed by two halves H_a and H_b of length l and m, respectively. Note that each half does not necessarily follow the chain connectivity (i.e. can contain bulged bases).
- Calculate the $\mathcal{E}$RMSD between H_a and all possible chain stretches of length l in a molecule.
- Create the collection of structures $\Gamma_a = \gamma_1, \gamma_2, \ldots$ such that $\mathcal{E}\text{RMSD}(H_a, \gamma) < C$ for each $\gamma \in \Gamma_a$. Typical values for the threshold C are between 0.5 and 1.0.
- Create the equivalent collection Γ_b obtained by calculating the $\mathcal{E}$RMSD with respect to H_b.
- Consider the cartesian product $\Gamma = \Gamma_a \times \Gamma_b$, that contains the set of pairs (γ_a, γ_b), where $\gamma_a \in \Gamma_a$ and $\gamma_b \in \Gamma_b$.
- Remove from Γ all pairs with sequence overlaps.
- Remove from Γ all pairs such that $|CoM(\gamma_a) - CoM(\gamma_b)| > 2.5 \times |CoM(H_a) - CoM(H_b)|$, where CoM is the center of mass calculated on the centroids.
- Calculate the $\mathcal{E}RMSD$ between (H_a, H_b) and all instances in Γ and retain those with $\mathcal{E}RMSD < C$.

References

[1] Das, R. and Baker, D. (2007) *Proc. Natl. Acad. Sci. U.S.A.* **104(37)**, 14664–14669.

[2] Bernauer, J., Huang, X., Sim, A. Y., and Levitt, M. (2011) *RNA* **17(6)**, 1066–1075.

[3] Pronk, S., Páll, S., Schulz, R., Larsson, P., Bjelkmar, P., Apostolov, R., Shirts, M. R., Smith, J. C., Kasson, P. M., van derSpoel, D., Hess, B., and Lindhal, E. (2013) *Bioinformatics* **29(7)**, 845–854.

[4] Tribello, G. A., Bonomi, M., Branduardi, D., Camilloni, C., and Bussi, G. (2014) *Comput. Phys. Commun.* **185(2)**, 604–613.

[5] Hornak, V., Abel, R., Okur, A., Strockbine, B., Roitberg, A., and Simmerling, C. (2006) *Proteins* **65(3)**, 712–725.

[6] Pérez, A., Marchán, I., Svozil, D., Sponer, J., Cheatham III, T. E., Laughton, C. A., and Orozco, M. (2007) *Biophys. J.* **92(11)**, 3817–3829.

[7] Banas, P., Hollas, D., Zgarbová, M., Jurecka, P., Orozco, M., Cheatham III, T. E., Sponer, J., and Otyepka, M. (2010) *J. Chem. Theory Comput.* **6(12)**, 3836–3849.

[8] Bussi, G., Donadio, D., and Parrinello, M. (2007) *J. Chem. Phys.* **126(1)**, 014101.

[9] Berendsen, H. J., Postma, J. P. M., vanGunsteren, W. F., DiNola, A., and Haak, J. (1984) *J. Chem. Phys.* **81(8)**, 3684–3690.

[10] Darden, T., York, D., and Pedersen, L. (1993) *J. Chem. Phys.* **98(12)**, 10089–10092.

[11] Hess, B., Bekker, H., Berendsen, H. J., and Fraaije, J. G. (1997) *J. Comput. Chem.* **18(12)**, 1463–1472.

[12] Ennifar, E., Nikulin, A., Tishchenko, S., Serganov, A., Nevskaya, N., Garber, M., Ehresmann, B., Ehresmann, C., Nikonov, S., and Dumas, P. (2000) *J. Mol. Biol.* **304(1)**, 35–42.

[13] Jorgensen, W. L., Chandrasekhar, J., Madura, J. D., Impey, R. W., and Klein, M. L. (1983) *J. Chem. Phys.* **79(2)**, 926–935.

[14] Parisien, M., Cruz, J. A., Westhof, É., and Major, F. (2009) *RNA* **15(10)**, 1875–1885.

[15] Serganov, A., Yuan, Y.-R., Pikovskaya, O., Polonskaia, A., Malinina, L., Phan, A. T., Hobartner, C., Micura, R., Breaker, R. R., and Patel, D. J. (2004) *Chemi. Biol.* **11(12)**, 1729–1741.
